# Exfoliative toxin E, a new *Staphylococcus aureus* virulence factor with host-specific activity

**DOI:** 10.1038/s41598-019-52777-3

**Published:** 2019-11-08

**Authors:** Ichiro Imanishi, Aurélie Nicolas, Ana-Carolina Barbosa Caetano, Thiago Luiz de Paula Castro, Natayme Rocha Tartaglia, Ricardo Mariutti, Eric Guédon, Sergine Even, Nadia Berkova, Raghuvir K. Arni, Nubia Seyffert, Vasco Azevedo, Koji Nishifuji, Yves Le Loir

**Affiliations:** 1 Laboratory of Veterinary Internal Medicine, Division of Animal Life Science, Graduate School, Tokyo University of Agriculture and Technology, 3-5-8 Saiwai-cho, Fuchu Tokyo, 183-8509 Japan; 2 STLO, INRA, Agrocampus Ouest, F-35042 Rennes, France; 3 Cellular and Molecular Genetics Laboratory, Institute of Biological Sciences, Federal University of Minas Gerais, Belo Horizonte, MG, 270-901 Brazil; 4 IBILCE/UNESP, São José do Rio Preto, SP, Brazil; 5 Institute of Health Sciences, Federal University of Bahia, Salvador, BA, 40110-100 Brazil; 6 Institute of Biology, Federal University of Bahia, Salvador, BA, 40170-115 Brazil

**Keywords:** Bacteriology, Pathogens

## Abstract

Exfoliative toxins (ETs) are secreted virulence factors produced by staphylococci. These serine proteases specifically cleave desmoglein 1 (Dsg1) in mammals and are key elements in staphylococcal skin infections. We recently identified a new *et* gene in *S. aureus* O46, a strain isolated from ovine mastitis. In the present study, we characterized the new *et* gene at a genetic level and the enzymatic activity of the deduced protein. The *S. aureus* O46 genome was re-assembled, annotated and compared with other publicly available *S. aureus* genomes. The deduced amino acid sequence of the new *et* gene shared 40%, 53% and 59% sequence identity to those of ETA, ETB and ETD, respectively. The new *et* gene shared the same genetic vicinity and was similar in other *S. aureus* strains bearing this gene. The recombinant enzyme of the new *et* gene caused skin exfoliation *in vivo* in neonatal mice. The new *et*-gene was thus named *ete*, encoding a new type (type E) of exfoliative toxin. We showed that ETE degraded the extracellular segments of Dsg1 in murine, ovine and caprine epidermis, as well as in ovine teat canal epithelia, but not that in bovine epidermis. We further showed that it directly hydrolyzed human and swine Dsg1 as well as murine Dsg1α and Dsg1β, but not canine Dsg1 or murine Dsg1γ. Molecular modeling revealed a correlation between the preferred orientation of ETE docking on its Dsg1 cleavage site and species-specific cleavage activity, suggesting that the docking step preceding cleavage accounts for the ETE species-specificity. This new virulence factor may contribute to the bacterial colonization on the stratified epithelia in certain ruminants with mastitis.

## Introduction

*Staphylococcus aureus* is a major Gram-positive pathogen and a serious threat to both human and animal health^1^ since it is implicated in numerous diseases ranging from superficial skin infections such as staphylococcal scalded skin syndrome (SSSS) to life-threatening endocarditis or sepsis in humans. *S. aureus* produces a wide array of virulence factors, which alone or in conjunction with other proteins contributes to the type and severity of staphylococcal infections. Most *S. aureus* virulence genes are borne by mobile genetic elements (MGE) and the type and severity of *S. aureus* infections therefore depends on strain-specific traits as much as on host traits. Although humans are the primary ecological niche and reservoir of *S. aureus*, it is also encountered in a variety of animal hosts. Livestock-associated strains have evolved following human-to-animal host jumps. This adaptive evolution has led to the emergence of endemic and sometimes host-restricted clones, and can be demonstrated at the genotype, genomic and molecular levels^2,3^. In dairy ruminants, *S. aureus* is a major causal agent of mastitis, an inflammation of the mammary gland that often results from a bacterial infection. Mastitis causes significant economic loss in the milk production chain. *S. aureus* strains isolated from ruminant hosts exhibit specific traits^4^ that might be useful in targeting and developing strategies for the prevention, or treatment of mastitis.

Exfoliative toxins belong to a family of serine proteases that display exquisite substrate specificity and recognize and hydrolyze a single peptide bond in the extracellular segment of desmoglein 1 (Dsg1), a desmosomal cadherin-type cell-cell adhesion molecule. This hydrolysis causes a dissociation of keratinocytes in human and animal skin. To date, three different ET serotypes (ETA, ETB and ETD) whose deduced amino acid sequences are similar to trypsin-like serine proteases have been identified in *S. aureus* and associated with staphylococcal skin infections such as SSSS or bullous impetigo in humans^5^. Exfoliation caused by ETs is described in many phylogenetically distant hosts, although with different degrees of susceptibility, which indicates host specificity^6^.

We previously characterized *S. aureus* strains isolated from severe or mild ovine mastitis at the genomic, proteomic and seroproteomic levels^7,8^. These studies led to the identification of staphylococcal secreted proteins which were specifically encountered in strains associated with mild mastitis in ewes^7^. One of these proteins was comparable in its amino acid primary sequence with the previously described *S. aureus* ETD. Of note was the fact that it harbored the typical catalytic site encountered in the other ET proteins described to date. This protein was tentatively referred to as an ETD-like protein, and its crystal structure was determined^9^.

In the present study, we addressed the issue of the exfoliative activity of the new ET and its host-specificity using *in vitro* and *in vivo* experiments as well as molecular docking.

## Results

### Similarity and genetic vicinity of the new *S. aureus* O46 *et* gene with other *et* genes

**Figure 1:**
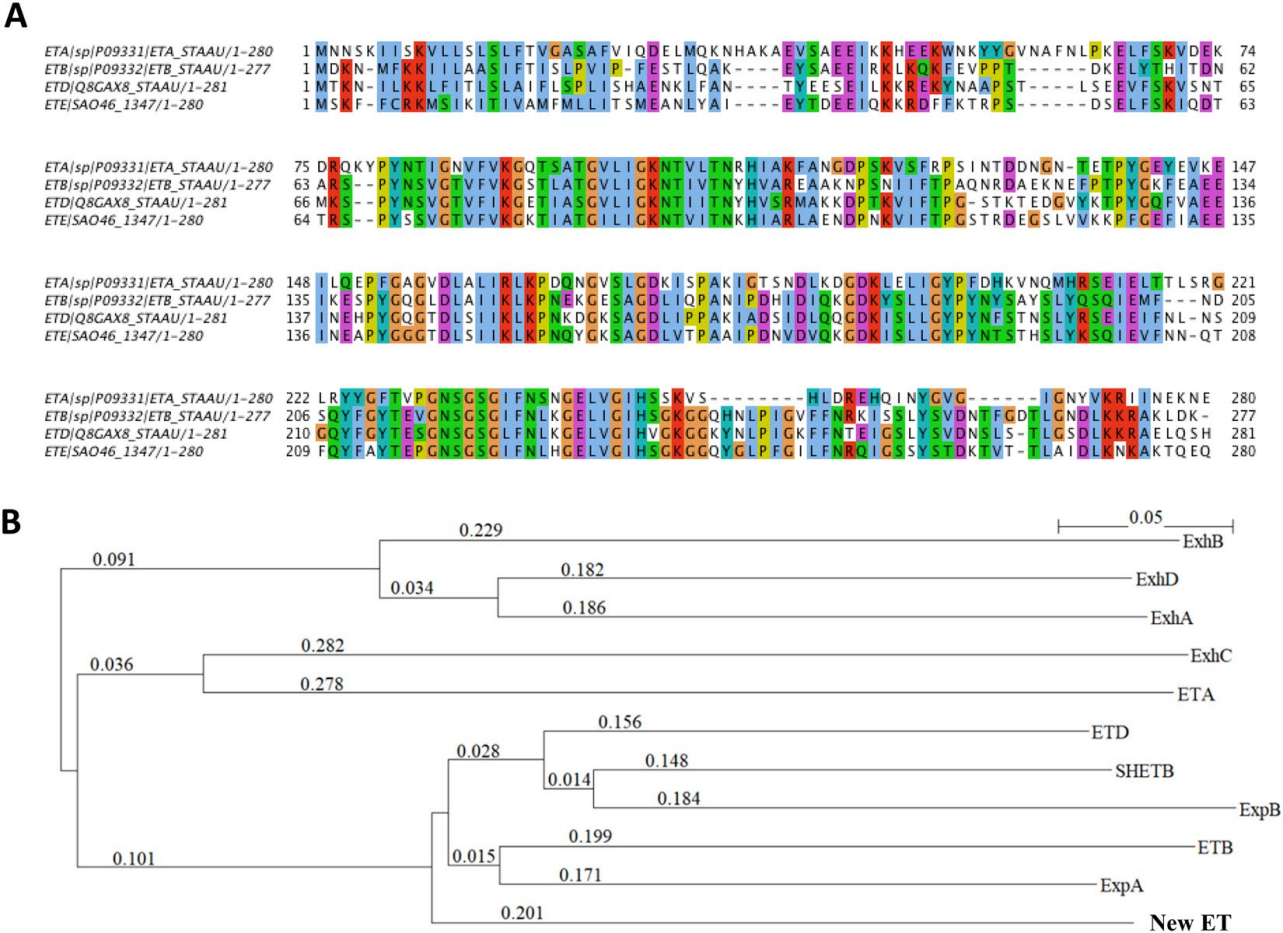
(**A**) Amino acid sequence alignment and phylogenetic tree of staphylococcal ETs. (**A**) Sequences retrieved from Uniprot were aligned with ClustalW with default parameters. (**B**) A phylogenetic analysis based on the overall amino acid sequences of ETs was built using a neighbor-joining method. SHETB, ExhA, ExhB, ExhC, and ExhD are ETs produced by *Staphylococcus hyicus*. ExpA and ExpB are ETs produced by *Staphylococcus pseudintermedius*. Three main clusters were observed, which group ExhA, ExhD and ExhB; ExhC and ETA; ExpA, ExpB, SHETB, ETB, ETD and the new ET (ETE).

**Figure 2:**
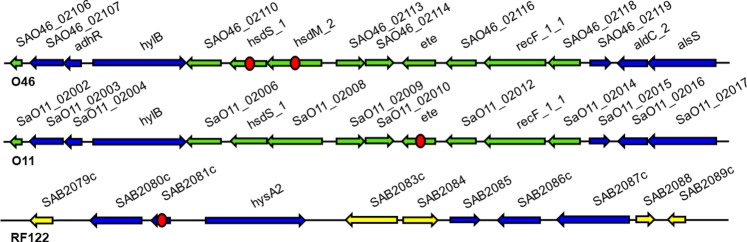
Putative genomic island containing the new *et* gene found in the *S. aureus* O46 genome. Comparisons with the most closely related putative genomic islands (GI) in strains O11 and RF122, isolated from ovine and bovine hosts, respectively, are shown below the upper line. Arrows represent open reading frames and their orientations. Blue: genes shared among O46, O11, and RF122 GIs. Green: genes shared between O46 and O11 GIs. Yellow: genes only present in the RF122 GI. Red circles indicate genes lacking part of their encoding sequence (*hsdM*_2 in O46, *ete* in O11) or presenting a frameshift that results in a coding sequence truncation (*hsdS*_1 in O46, *SAB2081c* in RF122).

The deduced amino acid sequence of the new *et* gene was compared with those of other characterized ET proteins in order to place this new protein in an ET phylogenetic tree (Fig. 1). The amino acid sequence of the new ET showed 59% sequence identity to Exfoliative toxin D (ETD)^10^, 40% sequence identity to ETA and 53% sequence identity to ETB (Fig. 1A)^11^. It clustered with those of *S. aureus* ETB and ETD, *Staphylococcus hyicus* SHETB^12^ and *Staphylococcus pseudintermedius* ExpA (EXI)^13,14^ and ExpB (Fig. 1B)^15^. A genomic analysis of strain O46 revealed eight putative genomic islands (GIs). The new *et* gene, along with 15 other genes, belongs to a 19.4 kb putative GI with a 30.8% GC content (i.e. lower than the average of 32.8% in the whole genome). This GI is not contained in any prophage regions of the chromosome. The features of the new *et*-containing GI are displayed in Fig. 2 and Table 1, while those of the genome region corresponding to putative GI, in strain RF122, are shown in Table S1. Multiple copies of Insertion Sequence (IS) families are present in the O46 genome (see Table S2); however, no IS was found in the genetic vicinity of the new *et*-containing GI region (2256079 to 2275534), which suggests that the GI is not part of a mobile genetic element.

**Table 1:** Genes present in the Putative Genomic Island of strain O46 containing the new *et* gene.

Gene name	Product/classification
sa_O462052	Hypothetical protein
sa_O462053	Oxidoreductase
*adhR*	Transcriptional regulator, MerR family
*hylB*	Hyaluronate lyase
sa_O462056	M23/M37 peptidase domain protein
*hsdS*_1	Type I restriction modification system, DNA specificity domain protein
*hsdM*_2	Type I restriction modification system, M subunit
sa_O462059	Serine protease
sa_O462060	Epidermal cell differentiation inhibitor
*ete*	Exfoliative toxin type E
sa_O462062	DNA helicase
*recF*_1_1	Recombinational DNA repair ATPase
sa_O462064	Hydrolase
sa_O462065	Probable exported protein
*aldC*_2	Alpha-acetolactate decarboxylase
*alsS*	Acetolactate synthase, catabolic

### Exfoliative activity of the new ET in neonatal mice

**Figure 3:**
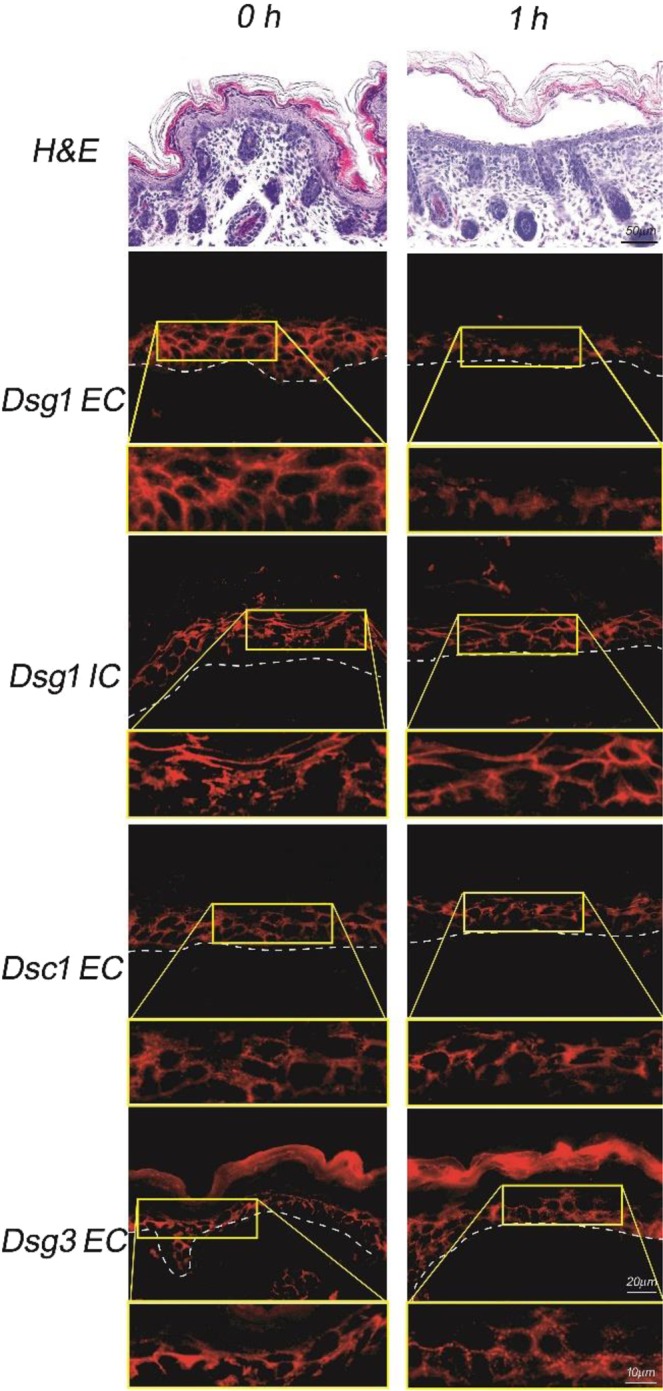
Exfoliative activity of the new ET in neonatal mice. Neonatal mice injected with a recombinant new ET displayed microscopic blisters 1 h after injection. Dotted lines indicate the basement membrane. Detailed views of microscopic blisters (areas delineated in yellow frame) are provided below each panel, at a higher magnification. Bars indicate 20 μm and 10 μm at lower and higher magnifications, respectively. EC: extracellular domain of Dsg, IC: intracellular domain of Dsg.

To determine whether the new ET exhibits exfoliative activity in the same way as classical ETs, the recombinant new ET was purified and injected to two neonatal mice. The skin sections obtained from those neonatal mice were subjected to histopathological and immunofluorescence analyses for Dsgs. Purity of the ETE sample was checked to confirm the absence of any other putative protease activity (see materials and methods for details). Histopathological analysis revealed intra-epidermal clefts with acantholysis in the stratum granulosum at the injection site as early as 1 h after injection (Fig. 3). Previous study reported that murine Dsg1α, Dsg1β and Dsg1γ show identical expression pattern in suprabasal layer of murine epidermis^16^. Immunofluorescence analysis revealed that IgG in a human pemphigus foliaceus (PF) serum, which mainly recognize the extracellular segments of murine Dsg1s^17^, was recognized in suprabasal epidermis. However, the IgG staining was markedly diminished from the cell-cell boundaries of the keratinocytes at the site wherein the new ET was injected (Figs 3 and S3). In contrast, those for the intracellular domain of Dsg1s and the extracellular segments of Dsc1 or Dsg3 were not affected (Figs 3 and S3). Altogether, these results clearly indicated that the new ET, which had previously been called ETD-like protein, share an enzymatic activity similar to that of known ETs. Therefore, the enzyme was renamed to ETE.

### ETE degrades Dsg1 in certain ruminants

**Figure 4:**
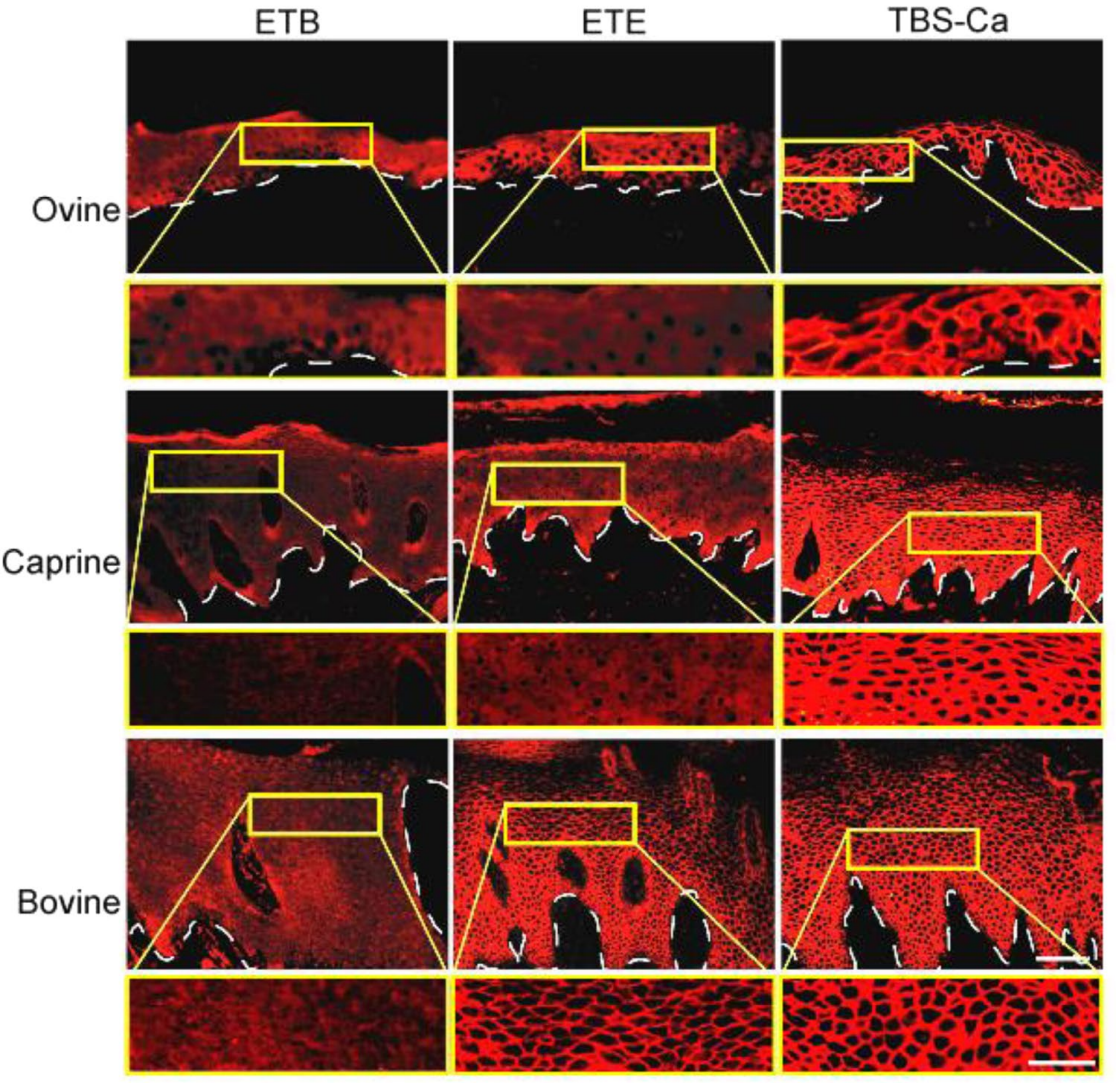
ETE degrades Dsg1 in ovine and caprine epidermis. Cryosectioned ovine, caprine and bovine nasal planum was incubated with ETB, the ETE protein or TBS-Ca, and subjected to immunofluorescence with human PF serum containing anti-Dsg1 IgG. Arrowheads indicate epidermal basement membranes. Detailed views of microscopic blisters (areas delineated in yellow frame) are provided below each panel, at a higher magnification. Bars indicate 50 μm and 20 μm at lower and higher magnifications, respectively.

To determine whether ETE affects Dsg1 in ruminants, purified recombinant ETE was incubated with cryosections of ovine, caprine and bovine nasal planum and underwent an immunofluorescence study with a human PF serum that recognize the extracellular segment of human^18^, murine and canine Dsg1^19^ (Figs 4 and S4). The IgG immunoreactivity in human PF serum against the cell-cell boundaries of the keratinocytes, which was recognized in suprabasal layers of the epidermis, was markedly diminished by the recombinant ETE in ovine and caprine nasal planum, but not in the bovine one. Recombinant ETB also diminished these immunoreactivities in all three ruminants tested. We repeated the same experiments twice and obtained identical results. Heat-inactivated ETE did not affect the immunofluorescence by the human PF serum (data not shown).

**Figure 5:**
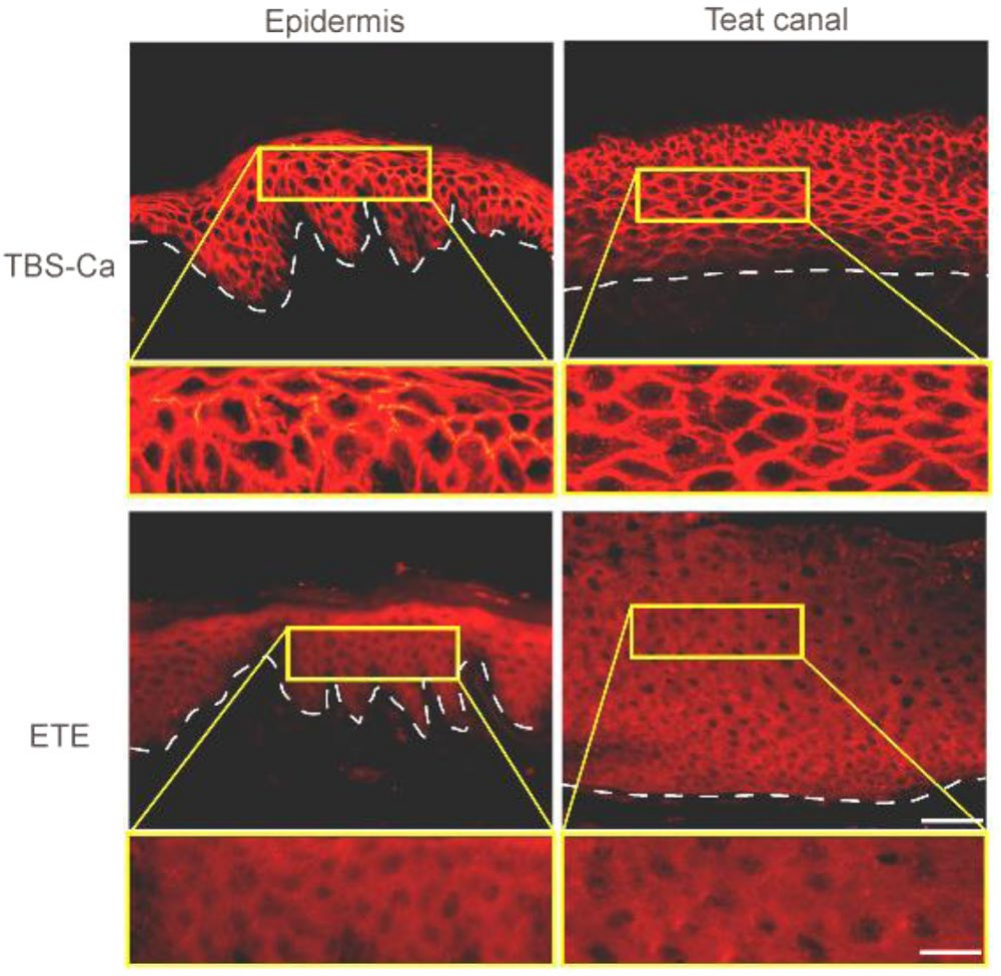
ETE degrades Dsg1 in caprine teat canal epithelia. Cryosections of caprine epidermis and teat canal were incubated with either TBS-Ca or ETE, and subjected to immunofluorescence with the human PF serum. Arrowheads indicate basement membranes. Detailed views of microscopic blisters (areas delineated in yellow frame) are provided below each panel, at a higher magnification. Bars indicate 50 μm and 20 μm at lower and higher magnifications, respectively.

Since *S. aureus* O46 was isolated in milk from an ewe with mastitis, we further investigated whether the target molecule for ETE is expressed in ruminant mammary glands or ducts. We found that the human PF IgG reacted with the plasma membrane of the whole layer in caprine teat canal epithelia, and IgG immunoreactivity to the cell-cell boundaries of the epithelial cells was markedly diminished when caprine teat canal cryosections were pre-incubated with recombinant ETE (Figs 5 and S5).

### ETE hydrolyzes the extracellular segments of Dsg1 in a species-specific manner

**Figure 6:**
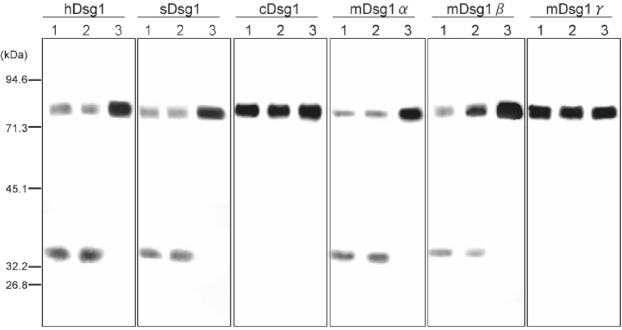
*In vitro* digestion of recombinant Dsg1s with ETE. Baculovirus recombinant extracellular domains of human Dsg1 (hDsg1), swine Dsg1 (sDsg1), canine Dsg1 (cDsg1), murine Dsg1α (mDsg1α), Dsg1β (mDsg1β) and Dsg1γ (mDsg1γ) were incubated with ETB (lane 1), ETE (lane 2), or TBS-Ca (lane 3), and subjected to immunoblotting with anti-E-tag monoclonal antibody. Original, non-cropped gel image is provided in the supplemental information file.

We further investigated whether ETE hydrolyzed Dsg1 in non-ruminants. As shown in Fig. 6, both recombinant ETE and ETB directly hydrolyzed the extracellular segments of human Dsg1 (hDsg1), swine Dsg1 (sDsg1), murine Dsg1α (mDsg1α) and mDsg1β into smaller peptides following *in vitro* incubation. The molecular weights of the degraded Dsg1 products produced by recombinant ETE were almost identical to those produced by ETB, suggesting that they recognize the same cleavage site. Conversely, neither ETE nor ETB degraded canine Dsg1 (cDsg1) or mDsg1γ. We repeated the same experiments twice and obtained identical results.

### Prevalence of the *ete* gene in other *S. aureus* genomes

The ETE predicted protein sequence was found in only 33 of the 9759 *S. aureus* predicted proteomes available on the PATRIC database (Table S3). A predicted ETE sequence was found in five out of nine *S. aureus* strains isolated from ovine samples, including one strain presenting a truncated ETE, and one out of 94 *S. aureus* strains isolated from bovine samples. Moreover, the predicted ETE protein was found in one strain isolated from a food sample, and in two strains from bulk cow’s milk. The 24 remaining strains were isolated from humans or unknown hosts. Six of the latter human strains were isolated in patients suffering from Buruli ulcers.

### Similarity of Dsg1 sequences and 3D structures in various mammalian species

**Figure 7:**
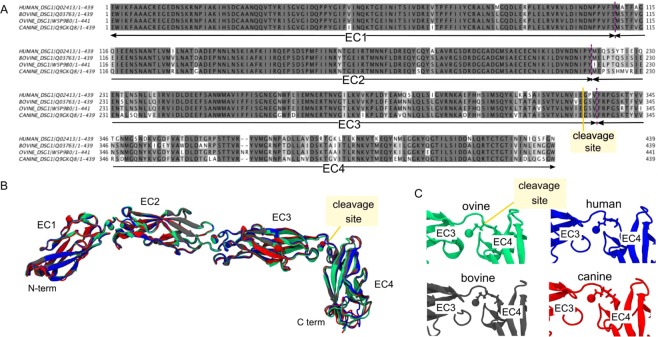
Protein sequence alignment and structural model of Dsg1. (**A**) Dsg1 protein sequence alignment of the extracellular domain. The boundaries of each cadherin repeat (EC1 to EC4) are indicated by a purple dashed line. The cleavage site is indicated by a yellow line. Residue numbers are according to model structures. (**B**) Superimposition of Dsg1 model structures. Ovine Dsg1 in green, human Dsg1 in blue, bovine Dsg1 in black and canine Dsg1 in red. Colored spheres are calcium ions. The cleavage site is indicated. (**C**) Zoom on cleavage site with the side chain of cleaved Glu332 represented and the unique calcium ion.

A comparison of amino acid sequences showed that glutamate residues cleaved by the well-characterized ETA, ETB, and ETD are conserved in ovine, human, bovine and canine Dsg1, whose amino acid sequences are available in the Uniprot database (N.B. the caprine Dsg1 sequence is not yet available), but some residues around the cleavage sites are not conserved (Fig. 7A). Model structures generated using SwissModel showed highly similar 3D structures in all the four mammalian species tested (Fig. 7B) with Cα-atoms Root Mean Square Deviations (RMSDs) ranging from 2.1 to 2.3 Å. More precisely, orientation of the glutamate residue cleaved by ETs was the same in all four species with their side chains oriented toward the calcium ion (Fig. 7C).

### Two types of docking orientation predicted

**Figure 8:**
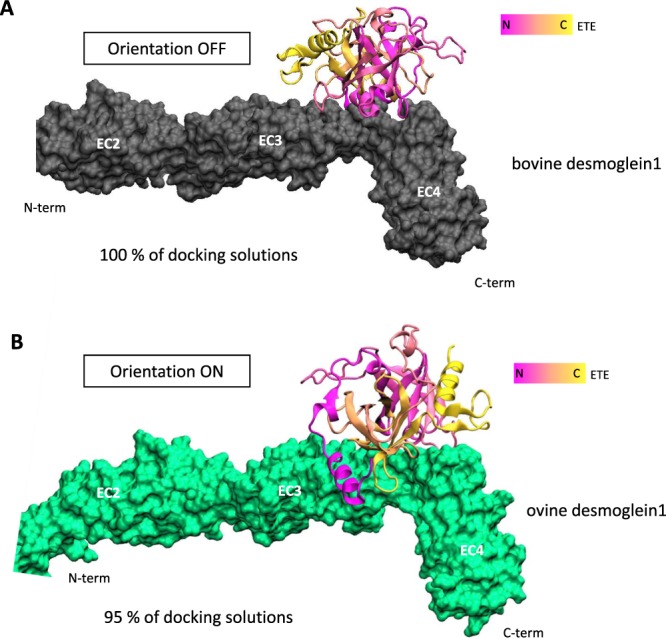
Best HADDOCK docking solutions of ETE on bovine and ovine Dsg1. ETE is colored from N terminus in pink to C terminus in yellow. (**A**) Orientation OFF (no cleavage) of ETE on bovine desmoglein (in black). (**B**) Orientation ON (cleavage) of ETE on ovine desmoglein (in green). For both bovine and ovine Dsg1, only EC2 to EC4 domains are shown.

Docking simulations of ETE with Dsg1 from four different species were performed using HADDOCK. Such molecular modeling approach allowed to investigate and to suggest molecular mechanisms involved in the host-specific cleavage of Dsg1. The software provides up to 200 docking solutions clustered by mean Root Mean Square Deviation (RMSD). All docking models of ETE on bovine Dsg1 are clustered in only one orientation with a score of −131.7 + /− 3.2 (Fig. 8A, Supplementary Table S4). Because bovine Dsg1 is not hydrolyzed by ETE, we named this orientation OFF. On the other hand, docking models of ETE on ovine Dsg1 are clustered in two orientations. The biggest cluster gathers 95% of docking models and is also energetically more favorable with a score of −147.1 +/− 1.3. It showed an opposite position of ETE on desmoglein (Fig. 8B, Supplementary Table S4). As ETE hydrolyzed ovine Dsg1, we named this orientation ON.

Docking simulations were also carried out with human and canine desmogleins (Supplementary Fig. S1) and displayed the same two ETE orientations. The biggest human docking model cluster was in orientation OFF but the more energetically favorable cluster was in orientation ON, as expected (Table S4, in supplemental data). The two best canine docking model clusters are in orientation OFF.

## Discussion

All *S. aureus* ETs are unique serine proteases that specifically and efficiently cleave a single peptide bond in the extracellular segment of Dsg1 in certain mammals. These properties cause efficient and specific abolishment of a major epidermal adhesion molecule in specific mammalian species. In the present study, we were able to characterize a new *S. aureus* ET that had initially been identified in *S. aureus* strains associated with mild mastitis in ewes^7^. It was previously named ETD-like protein because its predicted protein sequence determined from the O46 genome sequence displayed 59% identity with the ETD protein^7,9^. It also significantly differed from the other well-characterized ETA and ETB. Our work has clarified the fact that, despite those sequence divergence, this ETD-like protein has enzymatic activity similar to that of known ETs. This new ET protein indeed specifically cleaves the extracellular segment of murine Dsg1, but not that of Dsg3 or Dsc1. We are therefore proposing that this new *S. aureus* virulence factor should be renamed exfoliative toxin type E (ETE).

Like many other *S. aureus* toxins (e.g. staphylococcal enterotoxins), ETs are accessory proteins which are not essential for cell growth. The genes encoding these virulence factors in *S. aureus* are most often borne by mobile genetic elements (MGE) such as *S. aureus* pathogenicity islands (SaPI), prophages, transposons, and plasmids^6^. The genes encoding the well-documented ETA, ETB, and ETD proteins are indeed MGE-borne and carried by a temperate phage, a large plasmid, and a SaPI^20–22^, respectively. For the purposes of this study, the draft genome sequence of O46^23^ was re-assembled, re-annotated (GenBank accession number CP025395) and subjected to a search for genomic islands in order to obtain information on the genetic vicinity of *ete*. The *ete* gene was localized on one of the eight GIs that were predicted on the O46 genome. However, the *ete*-containing GI did not display any characteristic feature of MGE. Whether this results from horizontal transfer or recombination event remains unknown.

Genome data regarding *S. aureus* strains isolated from animal hosts remain scarce. To date only 343 *S. aureus* strains from animal hosts have been deposited on the PATRIC database whereas there are 6619 and 2797 genomes of *S. aureus* strains isolated from human or unknown hosts, respectively. However, out of the nine ovine strains available on PATRIC, four included a full ETE protein predicted in their proteomes, apart from one truncated ETE predicted in that of *S. aureus* O11. Although there might be a bias due to the low number of ovine strains, the proportion of 55.5% of *ete*-carrying strains is high, compared to only one out of the 94 bovine *S. aureus* proteomes (~1%) that include the ETE predicted protein. This observation correlates with our previous findings showing that the ETE protein is frequently (~half of all tested strains) detected in strains isolated from ewe mastitis^7^ and suggests that the *ete* gene is found more frequently in *S. aureus* strains isolated from ovine hosts than from any other hosts. Of note, the *ete* gene shared similar genetic vicinity in all the genomes of the strains isolated from ovine, bovine and bulk milk available on PATRIC. Dsg1, the ET substrate, is expressed in the teat canal epithelia of ruminants. Altogether, these data suggest that ETE provides ETE-producing *S. aureus* strains with advantage for the colonization of the mammary tract of certain ruminants with mastitis.

Although humans are the primary hosts for *S. aureus*, some of its lineages have evolved to adapt to a variety of animal hosts, and strains isolated from human or small (i.e. ovine or caprine) and large (i.e. bovine) ruminants have been shown to be distinct at both the genotypic and genomic levels^3^. Such species-specific traits are also observed at the molecular level. Indeed, certain virulence-associated genes have evolved towards species-specific activity, as illustrated with the bovine variant of von Willebrand factor-binding protein^24^, the equine variant of staphylococcal complement inhibitor (SCIN^25^), or ovine variants of staphylococcal enterotoxin type C^26^. The ETE described here displayed species-specific activity among ruminant Dsg1, with an efficient cleavage of ovine and caprine Dsg1, whereas it was not active on bovine Dsg1. Likewise, though all ETs are active against human Dsg1, some of them have evolved to develop activity against a broader array of hosts, *e.g*. *S. hyicus* Exhs which is active on swine Dsg1^27,28^.

All ETs are unique glutamate-specific serine proteases and specifically hydrolyze a peptide bond after glutamic acid residue 381, which is located between the extracellular domains 3 and 4^29^ of human and murine Dsg1(α). Mouse has three isoforms of Dsg1 (Dsg1α, Dsg1β and Dsg1γ)^16^. Among three isoforms of murine Dsg1, murine Dsg1α and Dsg1β are cleaved by ETA, ETB and ETD, while Dsg1γ in which catalytic glutamate residue 381 is replaced by lysine is unaffected^16,17^. In addition, the effects of ETA, ETB and ETD on keratinocyte dissociation and the formation of flaccid blisters in murine epidermis are histologically indistinguishable^10,30,31^. We showed that ETE hydrolyzed the extracellular segments of murine Dsg1α and Dsg1β, but not that of Dsg1γ. Moreover, molecular size of cleaved murine Dsg1α and Dsg1β by ETE were identical to those cleaved by ETB. These findings suggested that glutamic acid residue 381 is the site for the cleavage of Dsg1 by ETE.

Except for murine Dsg1γ, the ET cleavage site on Dsg1 was found to be highly conserved in Dsg1 of all the animal species used in this study. The species-specificity we observed here is therefore likely to rely on steps preceding the cleavage event and on the differential abilities of ETs to reach their cleavage site. Molecular docking experiments are widely used to determine the ability of a ligand to recognize and bind to its putative receptor(s). But to date it has never been applied to investigating the docking of a serine protease such as ETE on its cleavage site. The molecular docking of ETE with Dsg1 from different species suggests some keys to the approach mechanisms involved in this species-specific cleavage. Depending on the species of origin of Dsg1, ETE seems to have privileged orientations for interaction. Orientation OFF is observed particularly with bovine and canine Dsg1 that are not hydrolyzed by ETE, whereas orientation ON is observed with ovine and human best complexes. Interestingly, in our study, the dominant orientation ON for human and ovine Dsg1 correlated with the species-specific cleavage of Dsg1 seen during *in vitro* and *ex vivo* analyses, whereas orientation OFF for canine and bovine Dsg1 corresponded to an absence of detectable ETE cleavage in these mammalian species. Although both orientations may be observed for human Dsg1 (see Fig. S1 and Table S4 in supplemental material), it is worth noting that orientation OFF corresponds to a less energetically favorable docking. Further investigations using flexible docking and molecular dynamics experiments may help to clarify whether this fully determines actual cleavage specificity, or not.

In conclusion, ETE is a new *S. aureus* virulence factor that is frequently associated with ovine *S. aureus* isolates. It is also a new example of host specialization in *S. aureus*. Of note was the fact that the *ete* gene had previously been found in *S. aureus* strains associated with mild mastitis in ewes^6^. ETE may therefore be a marker of *S. aureus* virulence in ewe mastitis. Whether and how it provides selective advantages for *S. aureus* colonization and persistence in the udder still needs to be clarified, but this may be of considerable value to the diagnosis and control of mastitis.

## Experimental Procedures

### Genomic analysis of *Staphylococcus aureus* O46

The genome sequencing of *S. aureus* O46 and O11 was carried out by Maréchal *et al*^23^. using Solexa technology (Illumina, San Diego, CA, USA). DNA reads were re-evaluated in order to close all the sequence gaps previously observed in the genome and to facilitate identification of the genomic context of the new *et* gene. SPAdes version 3.9.1^32^ was used for the *de novo* assembly of O46 and O11 genomes. Scaffolding was performed using CONTIGuator version 2.7^33^ and the genome of *S. aureus* ED133 (an ovine strain of *S. aureus* (CP001996.1) retrieved from the National Center for Biotechnology Information (NCBI) databases) was used as a reference. The sequence gaps were then filled using FGAP^34^ and any remaining gaps were closed based on consensus sequences found against the genome of strain ED133, using CLC Genomics Workbench 7.0 (Qiagen, USA). The genomes of strains O46 and O11 were submitted to the RAST server for automatic annotation^35^. Genomic island predictions were performed using GIPSy version 1.1.2^36^ and the genome of *Staphylococcus warneri* strain SG1, retrieved from the NCBI databases, was used as a reference. Genomic island (GI) sequences and their gene products were curated manually using the UniProtKB database and Artemis^37^. To verify whether the GIs localized in prophage regions, the whole genomes were assessed using PHAST^38^. To identify the presence of insertion sequences (IS) in the O46 genome, it was submitted to the IS Finder online database that uses algorithms such as BLAST for IS identification^39^. To locate IS families through the genome, it was submitted to ISMapper, a mapping-based tool to identify the site and orientation of IS in bacterial genomes^40^. The newly assembled and annotated *S. aureus* O46 genome is deposited in GenBank (accession number CP025395).

The *ete* gene sequence was searched for in other *S. aureus* genome sequences using BLASTp in the PATRIC database with a e-value threshold of 0.01^41^.

### Phylogenetic tree of ETs

Phylogenetic analysis based on the amino acid sequences of ETs was performed with the CLUSTAL X program (http://www.clustal.org). A neighbor-joining tree was constructed using NJPlot software (http://doua.prabi.fr/software/njplot).

### Recombinant *ete* gene product

The gene corresponding to newly described ET protein was cloned and protein production was carried out in *Escherichia coli* host as previously described^9^. Briefly, the new *et* gene, further named *ete*, was amplified from *S. aureus* O46 genomic DNA and cloned into a pD441 expression vector. Optimized *ete* sequence with *E. coli* preferential codons was synthesized and cloned into pD441-NH expression vector by DNA2.0 (ATUM, Newark, CA). The plasmid pD441-NH:*ete* was transformed into CD43 (DE3) pLysS *E. coli* strain according to OverExpress™ Electrocompetent Cells kit (Lucigen, Middleton, WI) instructions for protein production and purification. Overnight culture of recombinant *E. coli* strain transformed with pD441-NH:*ete* was diluted 100-fold with fresh LB broth containing kanamycin (34 mg/mL) and incubated at 30 °C until the optical density (OD_600_) reached 0.5 and was subsequently induced with 0.2 mM IPTG for 16 hours at 20 °C. Induced cells were harvested by centrifugation and lysed by sonication and centrifuged at 15,000 g for 15 minutes. The supernatant was subjected to affinity chromatography using an immobilized nickel column (GE) under native conditions and further purified using a Superdex G75 10/300 GL column. Purity of the ETE protein was determined by SDS-PAGE gels (Fig. S2). DLS measurements on the purified ETE sample showed a single protein population (not shown).

### Recombinant ETB

A recombinant plasmid containing the *etb* gene fused with 6X His tag at the carboxyl terminus was a gift from Dr. Motoyuki Sugai, Hiroshima University^42^. The recombinant plasmid was transformed in BL21 (DE3) competent cells (Merck, Darmstadt, Germany). The recombinant ETB was harvested from the cytoplasmic soluble fraction of BL21, purified with TALON metal affinity resin (Takara Bio, Kusatsu, Japan) and dialyzed against phosphate-buffered saline (PBS).

### ETE activity on neonatal mice

Neonatal ICR mice (<12 h of age; Sankyo Labo Service, Tokyo, Japan) were injected subcutaneously with 100 µg of the purified ETE. Skin samples were collected 1 h after the injection and subjected to histopathological and immunofluorescence studies. All the experiments using mice had been ethically approved by The Animal Research Committee at Tokyo University of Agriculture and Technology (No. 25–69) and were performed in accordance with the International Guiding Principles for Biomedical Research Involving Animals.

### Immunofluorescence study on mouse skin

Cryosections of non-fixed neonatal mouse skin were stained using the following antibodies: a human pemphigus foliaceus serum containing IgG against the extracellular segment of Dsg1 (1:500 dilution; a kind gift from Dr. Masayuki Amagai, Keio University School of Medicine)^43^, AK18 anti-Dsg3 mouse monoclonal IgG antibody that recognizes the extracellular domain of Dsg3 (1:500 dilution; a gift from Dr. Masayuki Amagai)^44^, DG3.10 mouse monoclonal IgG antibody that reacts with the cytoplasmic domain of Dsg1 + 2 (Progen, Heidelberg, Germany) and a human IgA pemphigus serum containing IgA antibodies against the extracellular segment of desmocollin (Dsc) 1 (a gift from Dr. Masayuki Amagai)^45^. Fluorescence was captured and examined under a BX51 fluorescent microscope (Olympus Corp., Tokyo, Japan).

### Immunofluorescence study of ruminant skin

Fresh ovine skin and mammary duct were collected from a cadaver of an ewe bred at Tokyo University of Agriculture and Technology immediately after a natural death. Fresh caprine and bovine nasal planum were collected from cadavers after euthanization at Azabu University due to reasons unrelated to this experiment. Breeding of the sheep for the purpose of education and research was ethically approved by Faculty of Agriculture Field Science Center at Tokyo University of Agriculture and Technology (Np. 20190329). Euthanization of a goat and a cattle was ethically approved by The Azabu University Animal Experimentation Committee (No. 150917–3). Cryosections of those tissues were incubated with 100 µg/mL of either ETB or the ETE protein in TBS with 5 mM CaCl2 (TBS-Ca), or TBS-Ca alone for 2 hours at 37 °C. The same concentration of ETE was pretreated at 97 °C for 10 min and also used for the immunofluorescence study. The sections were then immunostained with a human pemphigus foliaceus (PF) serum containing IgG autoantibodies that recognize the extracellular segment of Dsg1.

### Quantitative analysis of the immunofluorescence for desmosomal cadherins

Quantitative analysis of the desmosomal cadherins in keratinocytes were performed as previously described (Figs S3, S4 and S5)^17^. Briefly, pixel intensities of immunofluorescent intensities of Dsg1-EC by human PF serum, Dsg1-IC, Dsc1-EC and Dsg3-EC along diagonal lines drawn arbitrarily on the epithelial cells were measured by ImageJ 1.8.0q software (https://imagej.nih.gov/ij). The distance between two cell-cell boundaries along the diagonal lines were divided into six areas. The areas #1 and #6 always include the cell-cell boundaries of the epithelial cells, whereas the areas from #3 to #5 always include cytoplasms. The pixel intensities of these molecules in the six compartment areas were calculated by MS Excel (Microsoft, Redmond, WA). Dunnett’s test was performed to compare the intensities among the compartment areas.

### *In vitro* digestion of Dsg1 by ETs

Insect culture supernatants containing the baculovirus recombinant extracellular domains of human Dsg1 (hDsg1), swine Dsg1 (sDsg1), canine Dsg1 (cDsg1), and murine Dsg1α (mDsg1α), Dsg1β (mDsg1β) and Dsg1γ (mDsg1γ), fused with E- and His-tags on their carboxyl termini, were gifts from Dr. Masayuki Amagai^17,19,43,46,47^. The recombinant Dsg1s were incubated *in vitro* with 70 µg/mL purified ETE or ETB for 2 hours at 37 °C. Dsg1s that remained intact or were degraded by ETs were detected using an anti-E-tag monoclonal antibody (GE Healthcare, Buckinghamshire, UK). Non-cropped, non-modified immunoblotting images for *in vitro* digestion of recombinant Dsg1 are presented in supplemental data (Fig. S3).

### Molecular modeling

Human, bovine, canine and ovine protein sequences of Dsg1 were retrieved from UniProtKB (Respective entry id: Q02413, Q03763, Q9GKQ8, W5P9B0). SwissModel^48–50^ was used to obtain 3D structural models of the extracellular domain. Each sequence was submitted to the software, and the human PDB structure of Dsg3 (5EQX) was used as a pattern. For each species, the best model was chosen and minimized using 2000 steps of steepest descent minimization under the YASARA software.

The structure of the new ETE previously published by Mariutti *et al*^9^. (PDB id: 5C2Z) was also submitted to 2000 steps of steepest descent minimization utilizing YASARA software (http://www.yasara.org/).

### Molecular docking

Protein-protein docking was performed using HADDOCK (High Ambiguity Driven protein DOCKing)^51^. Residues involved in the active sites of both proteins were required. The active ETE residues were those defined in^9^ (His96, Asp145 and Ser219) and those for Dsg1 were those defined in^52^ (Glu381, Gly382 in protein sequence matching Glu332, Gly333 in structural models).

HADDOCK generated up to 200 docking solutions clustered by mean Root Mean Square Deviation (RMSD), and a global score for in combined energies was calculated. Clusters are numbered according to their size and ranked according to a lowest score for the best cluster. Two best clusters for each species were conserved for further analyses. The visualization and image generation of the structures were performed using VMD (Visual Molecular Dynamics)^53^.

## Supplementary information

Supplementary Information
