# Author Correction: Exfoliative toxin E, a new *Staphylococcus aureus* virulence factor with host-specific activity

**DOI:** 10.1038/s41598-020-59360-1

**Published:** 2020-02-07

**Authors:** Ichiro Imanishi, Aurélie Nicolas, Ana-Carolina Barbosa Caetano, Thiago Luiz de Paula Castro, Natayme Rocha Tartaglia, Ricardo Mariutti, Eric Guédon, Sergine Even, Nadia Berkova, Raghuvir K. Arni, Nubia Seyffert, Vasco Azevedo, Koji Nishifuji, Yves Le Loir

**Affiliations:** 1 Laboratory of Veterinary Internal Medicine, Division of Animal Life Science, Graduate School, Tokyo University of Agriculture and Technology, 3-5-8 Saiwai-cho, Fuchu, Tokyo 183-8509 Japan; 2 STLO, INRA, Agrocampus Ouest, F-35042 Rennes, France; 3 Cellular and Molecular Genetics Laboratory, Institute of Biological Sciences, Federal University of Minas Gerais, Belo Horizonte, MG 270-901 Brazil; 4 IBILCE/UNESP, São José do Rio Preto, SP Brazil; 5 Institute of Health Sciences, Federal University of Bahia, Salvador, BA 40110-100 Brazil; 6 Institute of Biology, Federal University of Bahia, Salvador, BA 40170-115 Brazil

Correction to: *Scientific Reports* 10.1038/s41598-019-52777-3, published online 08 November 2019

In the Supplementary Information file originally published with this Article, supplementary figure S6, showing Best HADDOCK solutions for ETE docking on Dsg1 of four animal species, and supplementary table S4, showing HADDOCK scores, were omitted. These errors have been corrected in the Supplementary Information that now accompanies the Article.

Due to the omitted figure and table, there were additional errors in the main body of the Article.

In the Results section, under the subheading ‘Two types of docking orientation predicted’,

“Docking simulations were also carried out with human and canine desmogleins (Supplementary Fig. S1) and displayed the same two ETE orientations.”

now reads,

“Docking simulations were also carried out with human and canine desmogleins (Supplementary Fig. S6) and displayed the same two ETE orientations.”

In the Experimental Procedures section, under the subheading ‘Recombinant *ete* gene product’,

“Purity of the ETE protein was determined by SDS-PAGE gels (Fig. S2).”

now reads,

“Purity of the ETE protein was determined by SDS-PAGE gels (Fig. S1).”

Under the subheading ‘*In vitro* digestion of Dsg1 by ETs’,

“Non-cropped, non-modified immunoblotting images for *in vitro* digestion of recombinant Dsg1 are presented in supplemental data (Fig. S3).”

now reads,

“Non-cropped, non-modified immunoblotting images for *in vitro* digestion of recombinant Dsg1 are presented in supplemental data (Fig. S2).”

Additionally, there were typographical errors in the Experimental Procedures section, under the subheading ‘Molecular docking’,

“Residues involved in the active sites of both proteins were required. The active ETE residues were those defined in^9^ (His96, Asp145 and Ser219) and those for Dsg1 were those defined in^52^ (Glu381, Gly382 in protein sequence matching Glu332, Gly333 in structural models).”

now reads,

“Residues involved in the active sites of both proteins were required. The active ETE residues were those defined in Mariutti *et al*.^9^ (His96, Asp145 and Ser219) and those for Dsg1 were those defined in Hanakawa *et al*.^52^ (Glu381, Gly382 in protein sequence matching Glu332, Gly333 in structural models).”

