# Supplementary material for: Exfoliative toxin E, a new Staphylococcus aureus virulence factor with host-specific activity
Source: Sci Rep. 2019 Nov 8;9:16336. doi: 10.1038/s41598-019-52777-3 (PMC6841975; doi:10.1038/s41598-019-52777-3)
Supplement: Supplementary file 1 — Supplementary Information [file 41598_2019_52777_MOESM1_ESM.docx]

**Supplementary Information**

**Exfoliative toxin E, a new *Staphylococcus aureus* virulence factor with host-specific activity.**

Ichiro Imanishi^1^, Aurélie Nicolas^2^, Ana-Carolina Barbosa Caetano^3^, Thiago Luiz de Paula Castro^3,5^, Natayme Rocha Tartaglia^2,3^, Ricardo Mariutti^4^, Eric Guédon^2^, Sergine Even^2^, Nadia Berkova^2^, Raghuvir K. Arni^4^, Nubia Seyffert^3,6^, Vasco Azevedo^3^, Koji Nishifuji^1^**^¶^** and Yves Le Loir^2^**^¶^**

**Figure S1: SDS-PAGE of ETE protein preparation samples.**

Recombinant ETE was recovered from E. coli extract by affinity chromatography using an immobilized nickel column (GE) under native conditions. It was further purified using a Superdex G75 10/300 GL column. Purity of the ETE protein was determined by SDS-PAGE gels.

Non-cropped, non-modified image of the Coomassie blue-stained SDS PAGE of ETE samples at 3 consecutive purification steps.

Lane 1, molecular weight markers; Lanes 2-4, three fractions of purified ETE.

**
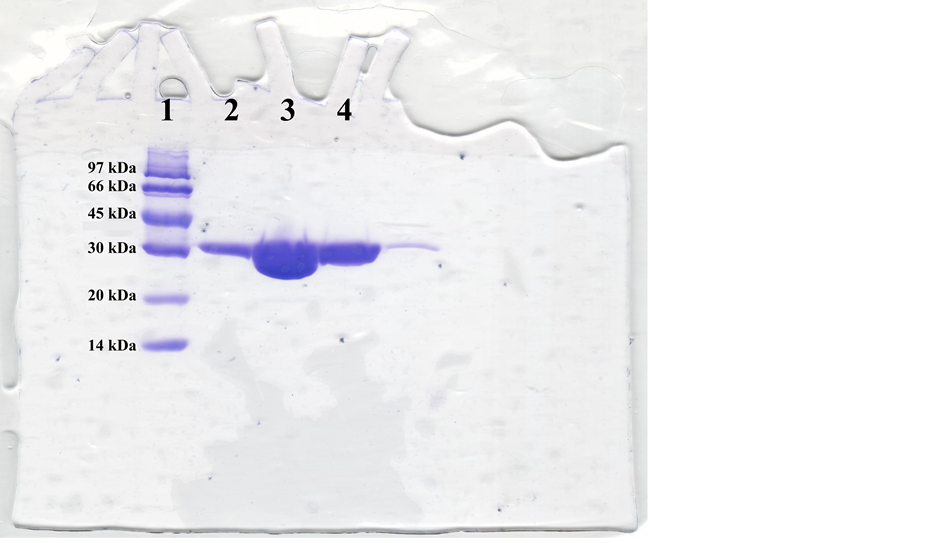
**

**Figure S2: Non-cropped, non-modified immunoblotting images for *in vitro* digestion of recombinant Dsg1s.**

Baculovirus recombinant extracellular domains of human Dsg1 (hDsg1), swine Dsg1 (sDsg1), canine Dsg1 (cDsg1), murine Dsg1α (mDsg1α), Dsg1β (mDsg1β) and Dsg1γ (mDsg1γ) were incubated with ETB (lane 1), ETE (lane 2), or TBS-Ca (lane 3), and subjected to immunoblotting with anti-E-tag monoclonal antibody. Representative blots were presented in Figure 6 (areas delineated in red frames).

**
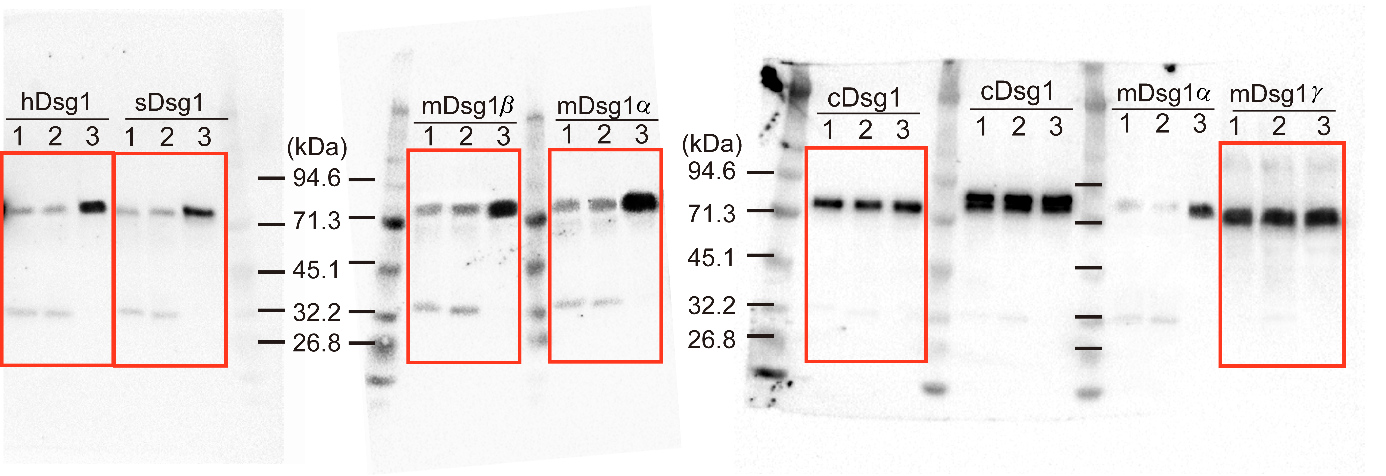
**

**Figure S3: Comparison of fluorescence intensities of desmosomal cadherins in murine keratinocytes.**

(A) Diagonal lines are drawn arbitrarily on murine keratinocytes and divided into six areas. The areas #1 and #6 include plasma cell boundaries. (B) Comparison of the fluorescent intensities of Dsg1-EC (extracellular segments), Dsg1-IC (intracellular segments), Dsc1-EC, and Dsg3-EC in six compartment areas of the epidermal keratinocytes before or 1h after the injection of ETE to neonatal mice. Graph shows the mean ± SD of pixel intensities of 30 diagonal lines one 10 keratinocytes. Asterisks indicate significantly different among the compartment areas of the keratinocytes (Dunnett's test, *P* < 0.05).


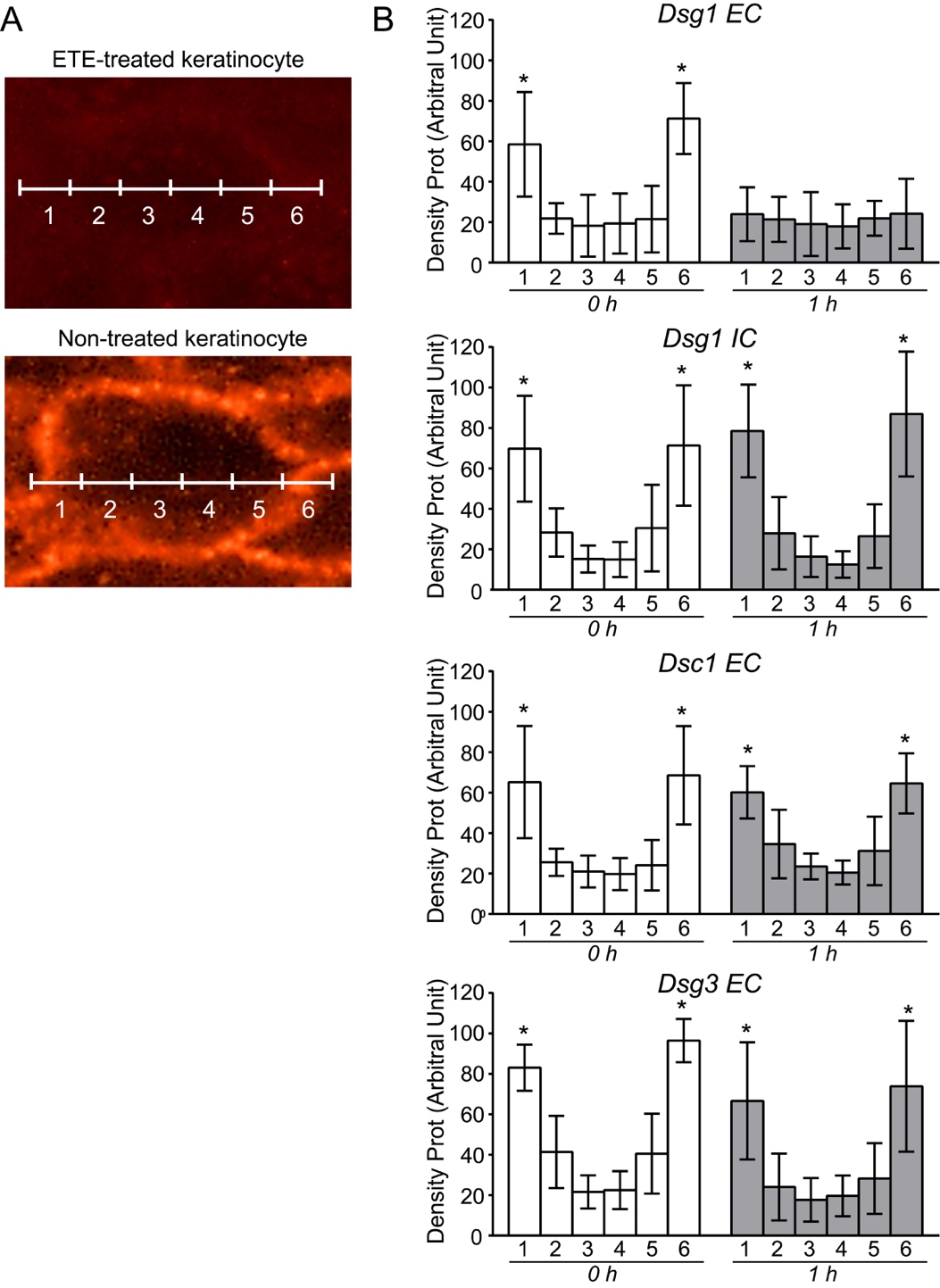


**Figure S4: Comparison of the fluorescent intensities of Dsg1-EC in ovine, caprine and bovine keratinocytes.**

Fluorescent intensities in the compartment areas 1 to 6 on diagonal lines drawn arbitrarily on ovine, caprine or bovine keratinocytes after the treatement with ETB, ETE or control (TBS-Ca) are compared. Graph shows the mean ± SD of pixel intensities of 30 diagonal lines in 10 keratinocytes. Asterisks indicate significantly different among the compartment areas (Dunnett's test, *P* < 0.05).





**Figure S5: Comparison of the fluorescent intensities of Dsg1-EC in keratinocytes derived from caprine epidermis and teat canal.**

Fluorescent intensities in the compartment areas 1 to 6 on diagonal lines drawn arbitrarily on the epithelial cells in ovine epidermis or teat canal after the treatement with ETE or control (TBS-Ca) are compared. Graph shows the mean ± SD of pixel intensities of 30 diagonal lines in 10 epithelial cells. Asterisks indicate significantly different among the compartment areas (Dunnett's test, *P* < 0.05).





**Table S1.** Genes present in the most closely related GI of strain RF122, relative to the ETE containing O46 SaPI.

| **Gene name** | **Product/ classification** |
| --- | --- |
| SAB2079c | Hypothetical protein |
| SAB2080c | Oxidoreductase |
| SAB2181c | Transcriptional regulator,  MerR family |
| hysA2 | Hyaluronate lyase |
| SAB2083c | Transcriptional regulator |
| SAB2084 | Conserved hypothetical protein |
| SAB2085 | Exported protein |
| SAB2086c | Alpha-acetolactate decarboxilase |
| SAB2087c | Alpha- acetolactate synthase |
| SAB2088 | Hypothetical protein |
| SAB2029c | Hypothetical protein |

**Table S2.** Localization of IS families in the O46 genome

| Localization of IS families | | |
| --- | --- | --- |
|  | Start of sequence | End of sequence |
| IS3 family | 1946138 | 1948102 |
| ISL3 family | 768457 | 770315 |
|  | 1747334 | 1749208 |
|  | 1852210 | 1854054 |

**Table S3**: *S. aureus* harboring the *ete* gene.

| **Genome Name** | **Isolation Source** | **Isolation Country** | **Host Name** |
| --- | --- | --- | --- |
| *Staphylococcus aureus O46* | subclinical ewe mastitis | France | Sheep, *Ovis aries* |
| *Staphylococcus aureus* O11 | gangrenous ewe mastitis | France | Sheep, *Ovis aries* |
| *Staphylococcus aureus* strain 04Hi | nasal | Tanzania | NA |
| *Staphylococcus aureus* strain 011Hii | nasal | Tanzania | NA |
| *Staphylococcus aureus* strain GKP136-11 | bulk milk | United Kingdom | NA |
| *Staphylococcus aureus* strain GKP136-21 | bulk milk | United Kingdom | NA |
| *Staphylococcus aureus* strain 3688STDY6124964 | clinical sample / spesis | Thailand | Human, *Homo sapiens* |
| *Staphylococcus aureus* strain CHUV_1 | skin (isolated from infected Eritrean and non-Eritrean patients) | Switzerland | Human, *Homo sapiens* |
| *Staphylococcus aureus* strain CHUV_8 | skin (isolated from infected Eritrean and non-Eritrean patients) | Switzerland | Human, *Homo sapiens* |
| *Staphylococcus aureus* strain CHUV_5 | skin (isolated from infected Eritrean and non-Eritrean patients) | Switzerland | Human, *Homo sapiens* |
| *Staphylococcus aureus* strain CHUV_2 | skin (isolated from infected Eritrean and non-Eritrean patients) | Switzerland | Human, *Homo sapiens* |
| *Staphylococcus aureus* strain CHUV_4 | skin (isolated from infected Eritrean and non-Eritrean patients) | Switzerland | Human, *Homo sapiens* |
| *Staphylococcus aureus* strain CHUV_6 | skin (isolated from infected Eritrean and non-Eritrean patients) | Switzerland | Human, *Homo sapiens* |
| *Staphylococcus aureus strain* CHUV_7 | skin (isolated from infected Eritrean and non-Eritrean patients) | Switzerland | Human, *Homo sapiens* |
| *Staphylococcus aureus* strain CHUV_3 | skin (isolated from infected Eritrean and non-Eritrean patients) | Switzerland | Human, *Homo sapiens* |
| *Staphylococcus aureus* strain BSAR58 | nasal | Denmark | Human, *Homo sapiens* |
| *Staphylococcus aureus strain* BSAR136_2 | wound | Denmark | Human, *Homo sapiens* |
| *Staphylococcus aureus* strain BSAR141_2 | nasal | Denmark | Cattle, *Bos sp*. |
| *Staphylococcus aureus* strain BSAR113_2 | nasal | Denmark | Sheep, *Ovis sp*. |
| *Staphylococcus aureus* strain BSAR57 | blood | Denmark | Human, *Homo sapiens* |
| *Staphylococcus aureus* strain BSAR112 | nasal | Denmark | Sheep, *Ovis sp*. |
| *Staphylococcus aureus* strain BSAR111_2 | nasal | Denmark | Sheep, *Ovis sp*. |
| *Staphylococcus aureus* strain BU_G1074_t4 | wound | Ghana | Human, *Homo sapiens* |
| *Staphylococcus aureus* strain *BU*_G0301_t8 | wound | Ghana | Human, *Homo sapiens* |
| *Staphylococcus aureus* strain BU_G1101_t2 | wound | Ghana | Human, *Homo sapiens* |
| *Staphylococcus aureus* strain BU_N17W_t2 | nose | Ghana | Human, *Homo sapiens* |
| *Staphylococcus aureus* strain BU_G0706B_t8 | wound | Ghana | Human, *Homo sapiens* |
| *Staphylococcus aureus* strain BU_G1001_t8 | wound | Ghana | Human, *Homo sapiens* |
| *Staphylococcus aureus* strain BB155 | nasal | Mali | Human, *Homo sapiens* |
| *Staphylococcus aureus* C00012787 | clinical sample | NA | Human, *Homo sapiens* |
| *Staphylococcus aureus* C00012788 | clinical sample | NA | Human, *Homo sapiens* |
| *Staphylococcus aureus* C00012789 | clinical sample | NA | Human, *Homo sapiens* |
| *Staphylococcus aureus* subsp. *aureus* strain SA-006 | food | Switzerland | NA |
